# Severe limitations of the FEve metric of functional evenness and some alternative metrics

**DOI:** 10.1002/ece3.6974

**Published:** 2020-12-21

**Authors:** Evsey Kosman, Samuel M. Scheiner, Hans‐Rolf Gregorius

**Affiliations:** ^1^ Institute for Cereal Crops Improvement Tel Aviv University Tel Aviv Israel; ^2^ Division of Environmental Biology National Science Foundation Alexandria VA USA; ^3^ Institut für Populations‐ und ökologische Genetik Göttingen Germany; ^4^ Abteilung Forstgenetik und Forstpflanzenzüchtung Universität Göttingen Göttingen Germany

**Keywords:** biodiversity, functional diversity, functional evenness, nearest‐neighbor distance

## Abstract

The metric of functional evenness FEve is an example of how approaches to conceptualizing and measuring functional variability may go astray. This index has several critical conceptual and practical drawbacks:

1. Different values of the FEve index for the same community can be obtained if the species have unequal species abundances; this result is highly likely if most of the traits are categorical.
2. Very minor differences in even one pairwise distance can result in very different values of FEve.
3. FEve uses only a fraction of the information contained in the matrix of species distances. Counterintuitively, this can cause very similar FEve scores for communities with substantially different patterns of species dispersal in trait space.
4. FEve is a valid metric only if all species have exactly the same abundances. However, the meaning of FEve in such an instance is unclear as the purpose of the metric is to measure the variability of abundances in trait space.

We recommend not using the FEve metric in studies of functional variability. Given the wide usage of FEve index over the last decade, the validity of the conclusions based on those estimates is in question. Instead, we suggest three alternative metrics that combine variability in species distances in trait space with abundance in various ways. More broadly, we recommend that researchers think about which community properties (e.g., trait distances of a focus species to the nearest neighbor or all other species, variability of pairwise interactions between species) they want to measure and pick from among the appropriate metrics.

## Graphical abstract

The index of functional evenness FEve has critical conceptual and practical drawbacks. We recommend not using FEve metric in studies of functional variability. Instead, we suggest three alternative metrics that combine variability in species distances in trait space with abundance in various ways. Different values of the FEve index for the same three communities.

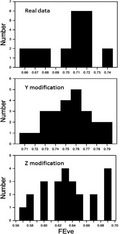

## 1 INTRODUCTION

Functional trait variability is a component of biodiversity that for the species within a community measures variability in the traits that are assumed to play a role in organismal or ecosystem functions. Many aspects of ecosystem processes depend on the nature, distribution, and variation of organismal traits. Therefore, a proper assessment of functional trait variability is important, and numerous metrics and approaches have been developed since the 1990s to measure this key community attribute (many of them are listed in Scheiner, 2019).

Biodiversity, of which functional trait variability is one component, is a complex concept. Scheiner et al. (2017b) pointed out that the three basic types of information—abundance, relatedness, and trait values—each have properties of magnitude and abundance. Together with species identity information (e.g., species richness), Scheiner (2019) defined fourteen basic elements of biodiversity that could be combined in myriad ways to produce many different types of biodiversity metrics. One scheme for describing different facets of functional trait variability was proposed by Villéger et al. (2008), who suggested three separate metrics: functional richness (FRic), functional evenness (FEve), and functional divergence (FDiv), which measure, respectively, the amount of trait space filled by the community, the evenness of species abundances as they are distributed in trait space, and how abundances are spread across trait space. In the classification scheme of Scheiner (2019), these are all composite metrics that, respectively, combine species richness with trait magnitude (FRic), abundance magnitude with trait variability of nearest‐neighbor distances (FEve), and abundance magnitude with trait variability of mean distances. Because they are composites and because of the way that trait magnitude and variability are measured, among the few commonly used approaches, these three metrics are some of the most complicated. They are assumed to provide an exhaustive measure of functional variability within a community, although that is clearly not the case given the limited types of information that they encompass. Despite some criticisms of these indices, mainly focused on functional evenness (e.g., Legras & Gaertner, 2018; Ricotta et al., 2014) and richness (e.g., Podani, 2009), their usage has continually grown in recent years from 134 citations in 2015 to 288 in 2019, with a current total of over 1,500 citations. In this paper, we demonstrate that functional evenness (FEve) has severe limitations in its applicability and interpretation. We concentrate on FEve as an example of how approaches to conceptualizing and measuring functional evenness may go astray.

A community can be characterized by its species and their abundances. Using additional information about those species, relationships among the species can be expressed in terms of pairwise distances that in turn can be used to measure overall community variation. In particular, if each species is described by the same set of *T* traits (standardized trait values are assumed), a community of *S* species can be represented by *S* points in a *T*‐dimensional trait space. While distances can be estimated with different metrics, relationships are completely predetermined by the species' dispersion in the trait space. Functional trait diversity can be measured in a variety of ways; the differences in trait space among species can be measured using all pairwise distances, the mean distance of a given species from other species, or nearest‐neighbor distances (Scheiner, 2019). Those distances can then be further weighted by the species abundances to provide a measure of abundance‐weighted functional trait variation within this multitrait space. FEve measures functional evenness based on abundance‐weighted nearest‐neighbor distances, so this metric might be relevant if the primary interactions within a community are among species that are most similar in trait values. While such types of interactions occur in many circumstances, there are many circumstances when this is not true for either species or types of interactions. However, the FEve metric has been widely used to analyze functional variation without consideration of the types of processes and entities being considered. We return to this issue in the final section of the paper when we discuss alternative measures of functional variation.

## 2 CONCEPTUAL PROBLEMS

Functional evenness (FEve) is based on a minimum spanning tree (MST) of a complete, undirected network of *S* vertices (species) with edges weighted by distance. An MST links all vertices through S‐1edges such that there are no cycles, that is, there is only one pathway between any two species. For *S* vertices, there are $S^{S‐2}$ possible spanning trees. The MST is the tree with the minimum possible total sum of the distances between all pairs of connected vertices (species). Importantly, several MSTs with the same minimum total distance may exist for a given network, if there are edges with the same distances. Such equal distances are highly likely if most of the traits are categorical or meristic (counts). At the extreme, if all edges are of equal distance, there are $S^{S‐2}$ MSTs.

If FEve is to be used as a measure of some property of biodiversity, its conceptual basis needs to be described and justified. In particular, what is the reasoning for the use of MST edges in combination with abundances as a functional characteristic? Which functional characteristic is addressed by this combination? In what sense is it a measure of evenness? In addressing these questions, we uncover two conceptual problems: (a) the possibility of nonuniqueness of MSTs and (b) its use as an index of evenness.

Given a particular MST with *S* nodes (species $s_{i}$, i=1,2,⋯,S), FEve is calculated as follows. First, each edge linking species $s_{i}$ and $s_{j}$ with functional distance $d_{\mathrm{ij}}=\mathrm{dist}\left(s_{i},s_{j}\right)$ between them is weighted by the sum of their abundances ($w_{i}$ and $w_{j}$):

(1) $${\text{EW}}_{\mathrm{ij}}=\frac{\text{dist}\left(s_{i},s_{j}\right)}{w_{i}+w_{j}}=\frac{d_{\mathrm{ij}}}{w_{i}+w_{j}}.$$

Second, those weighted edges are normalized by the sum of the ${\mathrm{EW}}_{\mathrm{ij}}$ values for the corresponding MST:

(2) $${\text{PEW}}_{\mathrm{ij}}=\frac{{\text{EW}}_{\mathrm{ij}}}{\sum_{(i,j)=1}^{S‐1}{\text{EW}}_{\mathrm{ij}}},$$

where $\left(i,j\right)$ designates an edge between species $s_{i}$ and $s_{j}$. (Because of this normalization, either relative or absolute abundances can be used.) Finally, FEve is calculated as follows:

(3) $$\text{FEve}=\frac{\sum_{(i,j)=1}^{S‐1}\min\left({\text{PEW}}_{\mathrm{ij}},\frac{1}{S‐1}\right)‐\frac{1}{S‐1}}{1‐\frac{1}{S‐1}},$$

which takes values between 0 and 1 (the denominator is the theoretically possible maximum value of the numerator). According to Villéger et al. (2008), "our new functional evenness index measures both the regularity of branch lengths in the MST and evenness in species abundances." From context, it is also clear that the authors intended the MST branches (edges) to connect nearest neighbors.

The authors do not explicitly identify the characteristics and objects that are the focus of their metric. We do so as follows. The combination of an edge plus abundances (PEW*_ij_* values) serves as the characteristic of interest, with pairs of species being the objects (Equation 1). Evenness of these objects (Equation 2) is the focus of the metric. Evenness is quantified as a deviation of the relative representations from their associated uniform distribution (the numerator in Equation 3).

This approach has several conceptual problems. First, abundance–edge combinations (EW*_ij_* values) do not necessarily represent evenness relationships between nearest neighbors. The internal nodes of an MST have at least two connecting edges. If one edge is smaller than another (e.g., $d_{\mathrm{ij}}<d_{\mathrm{ik}}$), its abundance‐weighted representation can be larger than that of the second edge ($E_{\mathrm{ij}}>E_{\mathrm{ik}}$) when the sum of abundances of the corresponding species is sufficiently larger ($w_{i}+w_{k}\gg w_{i}+w_{j}$). Depending on how species abundances are distributed along MST nodes, it is possible that none of those abundance–edge pairs on the MST represent nearest neighbors. Therefore, the estimation of functional evenness with FEve does not really mirror a concept of measuring functional variability based on the functionally nearest types (species).

Second, the authors state that "to transform species distribution in a T‐dimensional functional space to a distribution on a single axis, we choose to use the minimum spanning tree." No reasoning is given for why such a transformation is required. Nor is it explained in what way an MST can be considered as yielding a distribution on a single axis, given that nodes can connect to more than two others.

Third, the functional relevance of combining MST distances (edge values) with abundances is simply assumed. The use of abundances assumes that any and all functional traits have a similar per capita functional effect.

Fourth, the potential for a single set of points to have multiple MSTs is ignored. While one can demonstrate that the distribution of edge values is the same for all alternative MSTs, this property is lost when they are combined with species abundances. As we show in the next section, such combinations can lead to more than one value of evenness for the same data set.

## 3 INFERENCES FROM CONSTRUCTED EXAMPLES

**Figure 1:**
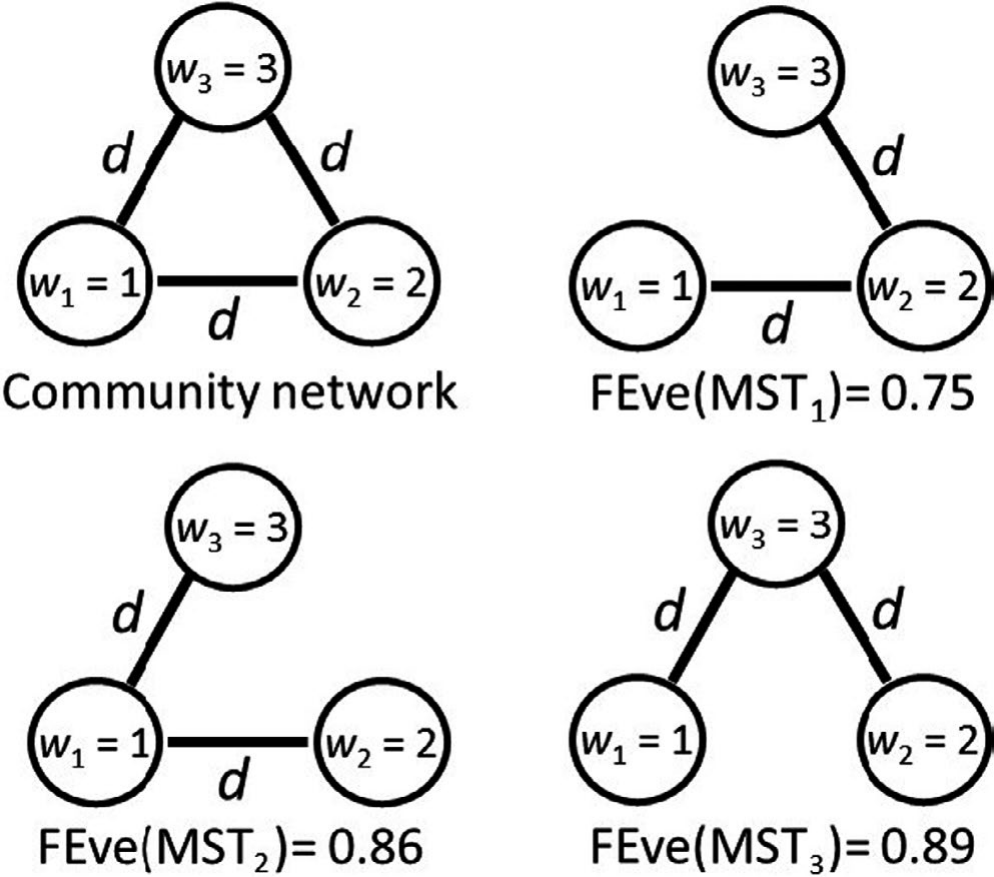
A community network in which the distances between all three species are identical, which results in three possible minimum spanning trees (MSTs) and multiple FEve estimates for the same community

Multiple MSTs can result in multiple, different values of FEve index for the same community if the species have unequal species abundances, which severely limit the utility of the metric. The following example demonstrates such a situation. Let the community consist of three equally distant species ($s_{1},s_{2}$, and $s_{3}$) in a given trait space (i.e., ${\mathrm{dist}\left(s_{1},s_{2}\right)=d}_{12}=d_{13}=d_{23}=d$) with abundances $w_{1}=1$, $w_{2}=2$, and $w_{3}=3$, respectively (Figure 1, community network). There are three MSTs with the same minimum total distance (2d): MST_1_ with one edge connecting $s_{1}$ and $s_{2}$, and one edge connecting $s_{2}$ and $s_{3}$ (Figure 1, MST_1_); MST_2_ with edges connecting $s_{1}$ and $s_{2}$, and $s_{1}$ and $s_{3}$ (Figure 1, MST_2_); and MST_3_ with edges connecting $s_{1}$ and $s_{3}$, and $s_{2}$ and $s_{3}$ (Figure 1, MST_3_). The three trees result in different estimates for FEve (Figure 1). Thus, for any community there is a likelihood for multiple FEve estimates making interpretation of any estimates suspect.

This problem does not arise if all distances for a given network are different; then, there will be only one, unique MST. Such differences in distances are likely if all or most of the traits are quantitative. However, such unique values for FEve do not solve the underlying conceptual problems.

Now consider the three MSTs in Figure 1 to be generated for three different communities and the distances between the species no longer identical, but just very, very slightly different so that each MST is unique for that community (e.g., for MST_1,_ *d*_12_ = *d*_23_ = 1 and *d*_13_ = 1.0001; for MST_2,_ *d*_12_ = *d*_13_ = 1 and *d*_23_ = 1.0001; for MST_3,_ *d*_13_ = *d*_23_ = 1 and *d*_12_ = 1.0001). Intuition says that the three communities have nearly the same evenness, and yet, they have very different values of FEve.

Additionally, hidden pitfalls come about from how FEve is often calculated. Rather than using the original matrix of pairwise distances, principal co‐ordinates analysis (PCoA) or multidimensional scaling (MDS) is used first to transform the distance matrix, and then, only the first two or three axes of the transformed space are considered when calculating species' distances (e.g., Mouillot et al., 2011; Taudiere & Violle, 2016). This transformation generally results in a distribution of nodes with no equal distances so that the corresponding MST is unique. However, because of the dimensional reduction, the new pairwise distances are only approximations of the original ones, and the corresponding FEve estimate depends on accuracy of PCoA performance (goodness of fit of the approximations to the original distances). While one could argue that the problems with FEve can be solved by always using untransformed distances, doing so does not guarantee a solution to the other problems listed above.

There is one circumstance that nonunique MSTs result in the same FEve values. This can happen if all species have exactly the same abundances. This equality occurs because any two MSTs of a given network have the same distribution of the edge weights. However, the meaning of FEve in such an instance is unclear as the purpose of the metric is to measure variability of abundances in trait space.

**Figure 2:**
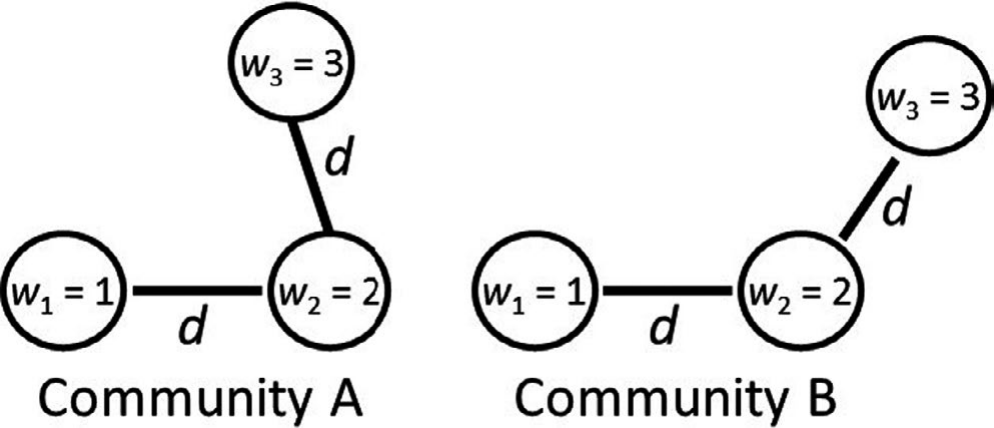
In these communities, two of the three species are equally distant in both communities (*d*_12_ = *d*_23_ = *d*) with a distance that is smaller than the third distance (*d*_13_). If the abundances of the three species are $w_{1}=1$, $w_{2}=2,$ and $w_{3}=3$, then FEve = 0.75, even though community B seems much more functionally irregular than community A

**Figure 3:**
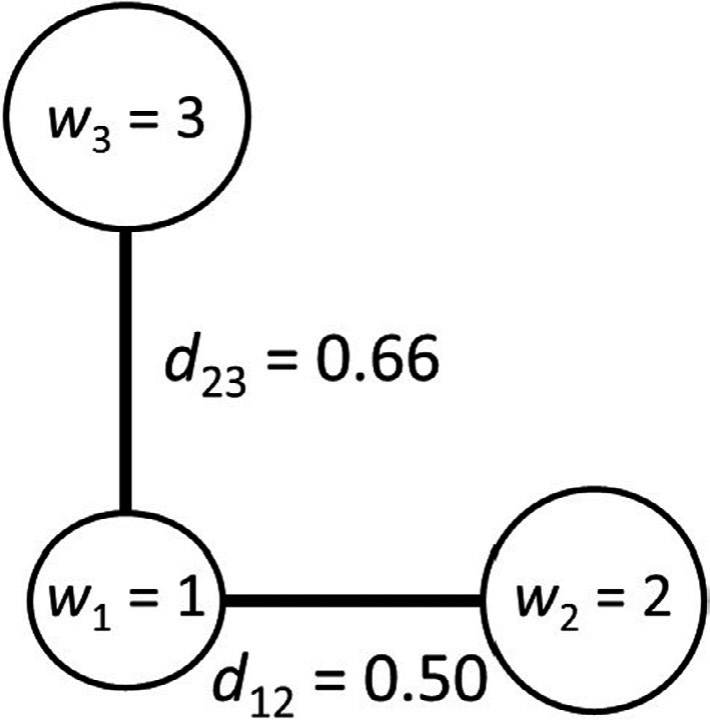
This community consists of three species in which *d*_23_ is larger than *d*_13_ and *d*_12_. The abundances (*w*) and distances result in values of ${\mathrm{EW}}_{12}={\mathrm{EW}}_{13}=1/6$, ${\mathrm{PEW}}_{12}={\mathrm{PEW}}_{13}=0.5$, and FEve = 1

A central reason for the problems raised above is that FEve uses only a fraction of the information contained in the matrix of species distances. Only S‐1 of the $\left(S‐1\right)\times S/2$ pairwise distances are used in the calculation of FEve; the much larger portion of the distances are simply ignored. This can cause the same FEve scores for communities with different patterns of species dispersal in trait space (Figure 2). In our example, this result occurs because the distance between species 1 and species 3 is ignored. In addition, it is possible to have a community where FEve = 1 even when neither species abundances nor distances between species are evenly distributed (Figure 3). In general, complete evenness (FEve = 1) is realized if and only if all ${\mathrm{PEW}}_{\mathrm{ij}}$ values are equal (Equations 2, 3), which does not necessarily imply that all distances or all abundances are equal. This behavior contradicts the claim of Villéger et al. (2008; p. 2293) that, “FEve decreases either when abundance is less evenly distributed among species or when functional distances among species are less regular.” Their claim is correct as an absolute statement only if the other factors (abundances or distances) are held constant, which will not occur when comparing actual communities.

## 4 EXAMPLES FROM DATA

Our constructed examples demonstrate the potential problems with the FEve metric. Here, we show how the problem of multiple estimates from a single dataset emerges with actual data. Importantly, there is no way to know in advance the number or range of different FEve estimates for a given dataset. Our first example is the traditional type of data used for functional trait analyses: bats and feeding traits. The other three examples are from less commonly used data: genetic profiles where the traits are the presence and absence of different genes. These examples demonstrate the problem of multiple MSTs that arises with noncontinuous traits. For the two examples that lack actual abundance data, we show how a single distance matrix can result in multiple, disparate FEve estimates with simulated abundances. For the other two examples, we show analyses with both actual abundances and two sets of simulated abundances to show how different types of abundance distributions can result in highly variable FEve estimates.

### 4.1 Bats and feeding traits

The first example consists of a set of five bat species (*Carollia manu*, *Chiroderma salvini*, *Dermanura glauca*, *Enchisthenes hartii*, and *Micronycteris megalotis*) in the Manu Biosphere Reserve located on the eastern slopes of the Andes in southeastern Peru. Our analysis was based on species characterization with 16 binary categorical traits (Table S3 in Scheiner et al., 2017a) that were separated into three groups: diet (fruit, nectar, invertebrates, vertebrates, fish, blood), foraging location (open areas, over water, above canopy, canopy, subcanopy, understory), and foraging strategy (aerial, gleaning, hovering, other). To determine the functional distance between species, Jaccard dissimilarity was calculated for each group of binary traits, and then, the combined distance between species was determined by an equal‐weight averaging of the three group‐specific dissimilarities (Table 1). Because the distance matrix contains many equal values, three different MSTs can be generated (Table 1). Because abundance data were not available, we provided two different sets of simulated values. For each set of simulated abundances, the multiple MSTs resulted in FEve estimates that varied 28% and 16%, respectively, between the smallest and largest values (0.374–0.480; and 0.676–0.785).

**Table 1:** For a set of five bat species: pairwise distances, abundances, MST attributes, and estimates of functional evenness FEve

		*B_1_*	*B_2_*	*B_3_*	*B_4_*	*B_5_*	Abundance^a^		MST edges	
*Carollia manu*	*B_1_*	0	0.333	0.333	0.333	0.167	***1***	20	*B_i_ – B_j_*	Weight
*Chiroderma salvini*	*B_2_*	0.333	0	0.167	0.222	0.5	***5***	20	1 – 5	0.167
*Dermanura glauca*	*B_3_*	0.333	0.167	0	0.167	0.5	***10***	20	1 – 2; 1 – 3; 1 – 4^b^	0.333
*Enchisthenes hartii*	*B_4_*	0.333	0.222	0.167	0	0.5	***20***	1	3 – 4	0.167
*Micronycteris megalotis*	*B_5_*	0.167	0.5	0.5	0.5	0	***1***	20	3 – 2	0.167

Note

Because no abundance data are available, we generated simulated values. The weights of the edges between each pair of MST nodes are equal to the distance between those species.

^a^ Two sets of simulated absolute abundances: Y (bold italic) and Z.

^b^ Because species *B_1_* is equally distant to three others, there are three possible MSTs. For set Y, the MSTs with edges 1‐2, 1‐3, and 1‐4 resulted in FEve estimates of 0.476, 0.480, and 0.374, respectively; for set Z, the values were 0.785, 0.785, and 0.676, respectively.

### 4.2 Bryozoan genotypes

*Cristatella mucedo* is a diploid freshwater bryozoan. We used data on eight microsatellite loci (Table 2 in Kosman & Jokela, 2019) for ten genetically separate individuals from bryozoan colonies in Lake Aegery, Switzerland. The distance between the genotypes was calculated by assuming a stepwise mutation model of microsatellite evolution with variable rates of mutations at different loci (SMMv; Kosman & Jokela, 2019). The corresponding matrix of pairwise distances is presented in Table 2. Abundance data were not available, so we provided simulated values. Again, multiple MSTs can be generated based on the distance matrix that results in four different FEve estimates (Table 2) that ranged from 0.533 to 0.635.

**Table 2:** Ten microsatellite genotypes of freshwater bryozoan *Cristatella mucedo* (Cm): pairwise distances, abundances, MST attributes, and estimates of functional evenness FEve

(A)	*Cm_1_*	*Cm_2_*	*Cm_3_*	*Cm_4_*	*Cm_5_*	*Cm_6_*	*Cm_7_*	*Cm_8_*	*Cm_9_*	*Cm_10_*	Abundance^a^	MST edges	
*Cm_1_*	0	0.066	0.021	0.009	0.066	0.052	0.054	0.078	0.083	0.124	***1***	*Cm_i_–Cm_j_*	Weight
*Cm_2_*	0.066	0	0.045	0.057	0.114	0.014	0.012	0.102	0.106	0.147	***2***	1–4	0.009
*Cm_3_*	0.021	0.045	0	0.012	0.069	0.059	0.057	0.057	0.062	0.103	***3***	4–3	0.012
*Cm_4_*	0.009	0.057	0.012	0	0.057	0.047	0.045	0.069	0.073	0.114	***4***	3–2; 4–7^b^	0.045
*Cm_5_*	0.066	0.114	0.069	0.057	0	0.104	0.102	0.012	0.016	0.057	***5***	2–7	0.012
*Cm_6_*	0.052	0.014	0.059	0.047	0.104	0	0.002	0.116	0.12	0.161	***6***	7–6	0.002
*Cm_7_*	0.054	0.012	0.057	0.045	0.102	0.002	0	0.114	0.118	0.159	***7***	4–5; 3–8^b^	0.057
*Cm_8_*	0.078	0.102	0.057	0.069	0.012	0.116	0.114	0	0.004	0.046	***8***	5–8	0.012
*Cm_9_*	0.083	0.106	0.062	0.073	0.016	0.12	0.118	0.004	0	0.05	***9***	8–9	0.004
*Cm_10_*	0.124	0.147	0.103	0.114	0.057	0.161	0.159	0.046	0.05	0	***10***	8–10	0.046

Note

Because no abundance data are available, we generated simulated values. The weights of the edges between each pair of nodes are equal to the distance between those genotypes.

^a^ Simulated absolute abundances.

^b^ There are four different MSTs with one of these edges. These MSTs resulted in four different FEve values: 0.533, 0.553, 0.612, and 0.635.

### 4.3 Wheat fungal pathogen (*Puccinia graminis* f. sp. tritici) genotypes

**Figure 4:**
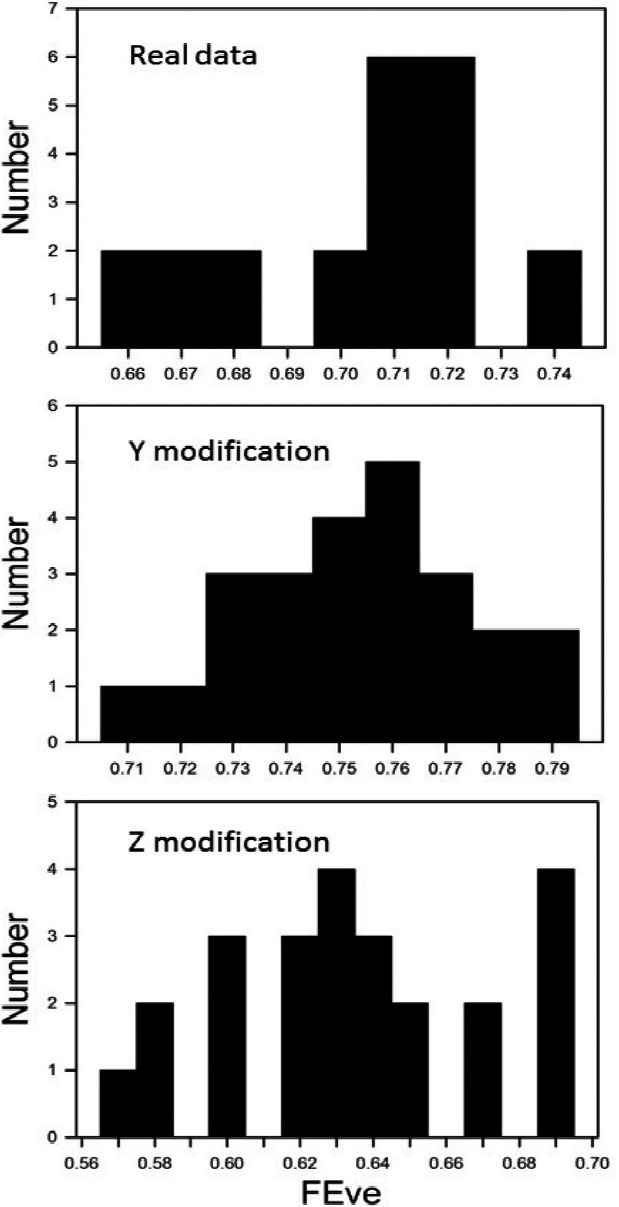
Variability of FEve estimates for the actual abundances of eleven virulence phenotypes of *Puccinia graminis*, and the Y‐modification and Z‐modification of abundances (see Table 3 for details)

The data consisted of eleven virulence phenotypes of *P. graminis* isolates collected from bread wheat in the Novosibirsk region of Russia. The binary phenotypes (virulence/avirulence) were determined with a set of twenty North American wheat differential lines (Skolotneva et al., 2020). The distance between the phenotypes was calculated using simple mismatch dissimilarity; the corresponding matrix of pairwise distances is presented in Table 3. Twenty‐four different MSTs can be generated (Table 3). For the actual abundances, ten different FEve estimates ranged from 0.659 to 0.737 (Figure 4). Even minor changes in abundances resulted in substantial changes in number and values of different FEve estimates: For the Y‐modification, twenty‐four values ranged from 0.708 to 0.793; for the Z‐modification, eighteen values ranged from 0.573 to 0.695 (Figure 4).

**Table 3:** Eleven virulence phenotypes of *Puccinia graminis* (Pgt): pairwise distances, abundances, MST attributes, and estimates of functional evenness FEve

	*Pgt_1_*	*Pgt_2_*	*Pgt_3_*	*Pgt_4_*	*Pgt_5_*	*Pgt_6_*	*Pgt_7_*	*Pgt_8_*	*Pgt_9_*	*Pgt_10_*	*Pgt_11_*	Abundance			MST edges	
*Pgt_1_*	0	0.05	0.1	0.15	0.1	0.1	0.15	0.05	0.15	0.3	0.45		***1***		*Pgt_i_* – *Pgt_j_*	Weight
*Pgt_2_*	0.05	0	0.05	0.1	0.15	0.15	0.2	0.1	0.1	0.35	0.4		***1***		1 – 2	0.05
*Pgt_3_*	0.1	0.05	0	0.05	0.2	0.1	0.15	0.15	0.15	0.3	0.35	(***3***)^a^	***2***	(***8***)^b^	2 – 3	0.05
*Pgt_4_*	0.15	0.1	0.05	0	0.15	0.15	0.2	0.2	0.1	0.35	0.3		***2***		3 – 4	0.05
*Pgt_5_*	0.1	0.15	0.2	0.15	0	0.2	0.15	0.15	0.15	0.3	0.35		***2***		1 – 8	0.05
*Pgt_6_*	0.1	0.15	0.1	0.15	0.2	0	0.15	0.15	0.25	0.2	0.35	(***4***)^a^	***1***		1 – 6; 3 – 6^c^	0.10
*Pgt_7_*	0.15	0.2	0.15	0.2	0.15	0.15	0	0.2	0.3	0.25	0.3		***2***		1 – 5	0.10
*Pgt_8_*	0.05	0.1	0.15	0.2	0.15	0.15	0.2	0	0.1	0.25	0.4		***9***		2 – 9; 4 – 9; 8 – 9^c^	0.10
*Pgt_9_*	0.15	0.1	0.15	0.1	0.15	0.25	0.3	0.1	0	0.35	0.3		***1***		1 – 7; 3 – 7; 5 – 7; 6 – 7^c^	0.15
*Pgt_10_*	0.3	0.35	0.3	0.35	0.3	0.2	0.25	0.25	0.35	0	0.25		***2***		6 – 10	0.20
*Pgt_11_*	0.45	0.4	0.35	0.3	0.35	0.35	0.3	0.4	0.3	0.25	0		***8***	(***2***)^b^	10 – 11	0.25

Note

The weights of the edges between each pair of nodes are equal to the distance between those phenotypes. To show the effects of variation in abundances, besides the actual values, we calculated FEve for each of two pairs of altered values.

^a^ Modification Y of abundances of two phenotypes.

^b^ Modification Z of abundances of two phenotypes; the total number of individuals was not changed.

^c^ Twenty‐four different MSTs are possible with one of these edges. The multiple MSTs resulted in ten, twenty‐four, and eighteen different FEve values for actual abundances, Y‐modification, and Z‐modification, respectively. Variability of FEve estimates is shown in Figure 4.

### 4.4 Wheat fungal pathogen (*Puccinia triticina* Erikss) genotypes

The data consist of eleven genotypes of single‐uredinial isolates of *P. triticina* (a dikaryotic fungus) collected from durum wheat in Russia using eleven microsatellite markers (Table 3 in Kosman & Jokela, 2019; Gultyaeva et al., 2017). The distance between the microsatellite genotypes was calculated assuming an infinite allele model (IAM; Kosman & Leonard, 2005), and the corresponding matrix of pairwise distances is presented in Table 4A. Three different MSTs can be generated based on the distance matrix (Table 4B). We compared the FEve estimates for the actual abundances with simulated values for three scenarios: (1) two dominant and nine rare types (simulation P), nine dominant and two rare types (simulation R), and all types equally abundant (simulation E). For the real abundances, FEve values ranged from 0.612 to 0.651 (about 7%). For simulation P, the values have a wider range (0.711–0.801, around 13%). For simulation R, the values have a very wide range, from 0.234 to 0.828 (about 354%), which shows the outsized influence of differences in MSTs when the node has a high abundance. For simulate E, as expected, equally abundant types resulted in the same value of 0.88 for all MSTs, despite their variation.

**Table 4:** (A) Eleven microsatellite genotypes of *P. triticina* (Pt): pairwise distances, abundances, and MST attributes

(A)	*Pt_1_*	*Pt_2_*	*Pt_3_*	*Pt_4_*	*Pt_5_*	*Pt_6_*	*Pt_7_*	*Pt_8_*	*Pt_9_*	*Pt_10_*	*Pt_11_*	Abundance			MST edges	
*Pt_1_*	0	0.045	0.045	0.091	0.045	0.136	0.091	0.091	0.136	0.182	0.182	***10*** ^a^	*1* ^b^	*20* ^c^	*Pt_i_ – Pt_j_*	Weight
*Pt_2_*	0.045	0	0.091	0.136	0.091	0.091	0.045	0.045	0.182	0.136	0.136	***12***	*1*	*20*	1 – 2	0.045
*Pt_3_*	0.045	0.091	0	0.136	0.091	0.182	0.045	0.136	0.091	0.136	0.227	***6***	*1*	*20*	1 – 3	0.045
*Pt_4_*	0.091	0.136	0.136	0	0.045	0.227	0.182	0.091	0.227	0.273	0.273	***1***	*1*	*20*	1 – 5	0.045
*Pt_5_*	0.045	0.091	0.091	0.045	0	0.182	0.136	0.045	0.182	0.227	0.227	***1***	*1*	*20*	5 – 4	0.045
*Pt_6_*	0.136	0.091	0.182	0.227	0.182	0	0.136	0.136	0.091	0.045	0.045	***2***	*1*	*20*	2 – 7	0.045
*Pt_7_*	0.091	0.045	0.045	0.182	0.136	0.136	0	0.091	0.136	0.091	0.182	***4***	*20*	*1*	2 – 8	0.045
*Pt_8_*	0.091	0.045	0.136	0.091	0.045	0.136	0.091	0	0.227	0.182	0.182	***1***	*1*	*20*	2 – 6; 3 – 9; 7 – 9^d^	0.091
*Pt_9_*	0.136	0.182	0.091	0.227	0.182	0.091	0.136	0.227	0	0.045	0.136	***1***	*20*	*1*	6 – 10	0.045
*Pt_10_*	0.182	0.136	0.136	0.273	0.227	0.045	0.091	0.182	0.045	0	0.091	***4***	*1*	*20*	10 – 9	0.045
*Pt_11_*	0.182	0.136	0.227	0.273	0.227	0.045	0.182	0.182	0.136	0.091	0	***1***	*1*	*20*	6 – 11	0.045

(B) MST	Abundances			
	Actual	Simulated P	Simulated R	Equal
1 (2–6)^d^	0.651	0.801	0.828	0.880
2 (3–9)	0.631	0.724	0.753	0.880
3 (7–9)	0.612	0.711	0.234	0.880

Note

The weights of the edges between each pair of nodes are equal to the distance between those genotypes. To show the effects of variation in abundances, besides the actual values, we calculated FEve for two instances of altered values

(B) Estimates of functional evenness FEve.

^a^ Actual abundances (bold italic).

^b^ Simulation P of abundances (italic).

^c^ Simulation R of abundances (italic underline).

^d^ There are three different MSTs with one of these edges.

## 5 SUMMARY OF FEVE ISSUES

In constructing species networks, it is assumed that trait values are measured without error and that there is no variation within species, two assumptions that we know are false. This issue could be addressed by a procedure that would estimate the mean and variability of relevant estimates over all closely related networks. We do not know of any attempt to study that matter for any diversity metric. Nevertheless, this problem seems much more acute for FEve compared with many other metrics because of the potential for nonunique MSTs in addition to errors in distance measures. Multiple estimates of a diversity metric obtained for different, closely related networks are natural. However, it is conceptually incorrect to assume that functional evenness has multiple values for a community represented by a single network. Given the wide usage of FEve index over the last decade, the validity of the conclusions from those studies is now in question. Our examples show that a single dataset can result in considerable variability in FEve estimates, especially when the data include rare types. The combination of functional relationships and abundances (species distance divided by sum of their abundances) into a single assessment of evenness results in a metric that fails to distinguish between distance evenness and abundance evenness (Gregorius, 1990).

This entire paper has been about FEve, but we would be remiss if we do not mention PEve—phylogenetic evenness—which was defined by Dehling et al. (2014) to be identical to FEve, but substituting nearest‐neighbor phylogenetic distances for distances in functional trait space. We discussed the nonuniqueness problem with FEve that occurs when you have two species which have identical nearest‐neighbor distances to a third, but differ in their abundances. This problem is most likely for categorical traits or those based on counts with just a few possible values and so many not occur that often. However, this problem is highly likely for phylogenetic data. It will occur any time you have a pair of sister species that are equally distant from a third and that differ in their abundances. PEve has been used much less frequently than FEve, but should also be abandoned. As with functional traits, there are alternatives for phylogenetic evenness that can measure the same properties while avoiding the uniqueness problem (Scheiner, 2019; Tucker et al., 2017).

## 6 NEXT STEPS

We have shown that FEve has critical conceptual and practical drawbacks, and therefore, we recommend not using this index in studies of functional variability. However, it is still possible to measure evenness of functional traits combined with information about abundances using alternative methods that do not have the limitations of FEve. An alternative metric based on Hill numbers that combines nearest‐neighbor distances with abundances is the evenness derivative of the diversity metric of Scheiner (2012):

(4) $${}^{q}D\left({\text{AT}}_{N}\right)={\left(\sum_{i=1}^{S}{\left(\frac{n_{i}d_{i\;\min}}{\sum_{j=1}^{S}n_{j}d_{j\;\min}}\right)}^{q}\right)}^{1/(1‐q)}$$

(5) $${}^{q}E\left({\text{AT}}_{N}\right)={}^{q}D\left({\text{AT}}_{N}\right)/S,$$

where ${}^{q}D\left({\text{AT}}_{N}\right)$ is effective number of distinct species that equally contribute to functional interaction and variability within a community based on nearest‐neighbor distances ($n_{i}d_{i\;\min}=n_{j}d_{j\;\min}$ for all i≠j), *S* is the number of species, *n_i_* is the number of individuals of species *i*, *d_i_*_min_ is the nearest‐neighbor distance of species *i*, and *q* is the exponent of the Hill function. [The metrics here and below follow the symbol convention of Scheiner (2019).] This metric measures the evenness of the joint distribution of abundances and nearest‐neighbor distances. Because each species has a unique nearest‐neighbor distance, the resulting metric always has a single value and small deviations of those values will result in only small changes in the metric, eliminating the problems that we outlined above for FEve. It should be used in conjunction with an examination of the separate evennesses of abundances [${}^{q}E\left(A\right)$] and nearest‐neighbor distances [${}^{q}E\left(T_{N}\right)$]. For example, it is possible that neither parameter is evenly distributed singly, but that the joint distribution has an even distribution, which can occur if the two components are strongly negatively correlated. Such a combination of values would then point to the potential importance of processes that jointly affect traits and abundances (e.g., competitive exclusion).

An alternative approach for combining trait distance and abundance information is the use of the abundance‐weighted distance of species *i* from all of the other S‐1 species:

(6) $$d_{i}=\sum_{\begin{array}{c} k=1\\ k\neq i \end{array}}^{S}d_{\mathrm{ik}}\left(n_{k}/N\right),$$

where $N=\sum_{j=1}^{S}n_{j}$ is the total number of individuals in the assemblage. Then, functional diversity can be estimated in terms of Hill numbers as follows:

(7) $${}^{q}D\left({\text{AT}}_{T}\right)={\left(\sum_{i=1}^{S}{\left(\frac{n_{i}d_{i}}{\sum_{j=1}^{S}n_{j}d_{j}}\right)}^{q}\right)}^{1/\left(1‐q\right)},$$

which is the effective number of distinct species that equally contribute to functional interaction and variability within a community based on abundances and weighted distances of every species from all other species ($n_{i}d_{i}=n_{j}d_{j}$ for all i≠j). From this, we can obtain an evenness measure as follows:

(8) $${}^{q}E\left({\text{AT}}_{T}\right)={}^{q}D\left({\text{AT}}_{T}\right)/S.$$

This metric is a Hill‐based generalization of the metrics of Guiasu and Guiasu (2012) and Ricotta et al. (2014). This measure of evenness would be appropriate if a given species interacts with all of the other species in a community in a way that "averages" over all of those interactions (e.g., in a system with diffuse competition).

The evenness metrics given in Equations 5 and 8 are based on the individual properties of each species. An alternative approach is to measure functional variation based on pairs of species:

(9) $${}^{q}H\left({\text{AT}}_{P}\right)={\left(\sum_{i=1}^{S}\sum_{j=1}^{S}{\left(\frac{n_{i}n_{j}d_{\mathrm{ij}}}{\sum_{k=1}^{S}\sum_{l=1}^{S}n_{k}n_{l}d_{\mathrm{kl}}}\right)}^{q}\right)}^{1/\left(1‐q\right)},$$

which measures the effective number of equally interacting pairs of species (equal values of $n_{i}n_{j}d_{\mathrm{ij}}$ for all i,j=1,2,⋯,S, i≠j) (see eq. A23 in Scheiner et al., 2017), so that the number of equally interacting species is determined as follows:

(10) $${}^{q}D\left(AT_{P}\right)=\left(1+\sqrt{1+4^{q}H\left(AT_{P}\right)}\right)/2,$$

(Equations 4 and A4, Scheiner et al., 2017). The corresponding metric of functional evenness is then as follows:

(11) $${}^{q}E\left({\text{AT}}_{P}\right)={}^{q}D\left({\text{AT}}_{P}\right)/S.$$

This measure of evenness would be appropriate if the pairwise interactions are important and those interactions occur with all of the other species in the community (e.g., scramble competition for a spectrum of resources). The metrics presented here (Equations (4), (5), (6), (7), (8), (9), (10), (11)), as well as FEve itself, assume that all individuals within a species are identical; somewhat different forms are necessary to capture within‐species variation.

More general concepts (Gregorius & Kosman, 2017, 2018) and a large variety of metrics (Scheiner, 2019) exist for measuring functional variation and can be used as alternatives for FEve. We caution, though, that many of them have not yet been critically evaluated. The metrics suggested here (Equations 5, 8, and 11) are all based on a concept of diversity of dispersion measured by an effective number of types. Division of this effective number by the actual number of types turns these into metrics of functional evenness. While there is no single best way to measure functional trait evenness or its combination with abundance, there are metrics, such as FEve, that should be avoided.

## CONFLICT OF INTEREST

None declared.

## AUTHOR CONTRIBUTIONS

**Evsey Kosman:** Conceptualization (lead); formal analysis (lead); funding acquisition (lead); investigation (equal); methodology (equal); visualization (supporting); writing–original draft (lead); writing–review and editing (equal). **Samuel Scheiner:** Conceptualization (supporting); investigation (equal); methodology (supporting); visualization (lead); writing–original draft (supporting); writing–review and editing (lead). **Hans‐Rolf Gregorius:** Conceptualization (supporting); formal analysis (supporting); investigation (equal); methodology (supporting); writing–review and editing (equal).

## Data Availability

All of the data used in this paper were previously published.
